# Physical activity and cognitive function: A comparison of rural and urban breast cancer survivors

**DOI:** 10.1371/journal.pone.0284189

**Published:** 2023-04-13

**Authors:** Lindsey L. Page, Christina J. Kahn, Joan Severson, Arthur F. Kramer, Edward McAuley, Diane K. Ehlers

**Affiliations:** 1 Department of Neurological Sciences, University of Nebraska Medical Center, Omaha, NE, United States of America; 2 Digital Artefacts, LLC, Iowa City, IA, United States of America; 3 Northeastern University, Boston, MA, United States of America; 4 University of Illinois at Urbana-Champaign, Urbana, IL, United States of America; 5 Mayo Clinic Arizona, Scottsdale, AZ, United States of America; The John Paul II Catholic University of Lublin, POLAND

## Abstract

**Purpose:**

Increasing evidence suggests rural breast cancer survivors (BCS) may experience greater burden in symptoms known to be associated with cancer-associated cognitive decline (CACD). Yet, little is known about CACD in rural BCS. This study (1) examined differences in cognitive function, moderate-to-vigorous physical activity (MVPA), and other CACD correlates and (2) tested the effects of MVPA on cognitive function in rural versus urban BCS.

**Methods:**

Rural and urban BCS (N = 80), matched on age, education, and time since diagnosis from a larger study, completed cognitive tasks assessing processing speed (Trails-B, Mazes, Task-Switch) and working memory (spatial working memory) and questionnaires assessing subjective memory impairment (SMI), MVPA, and CACD correlates (i.e., sleep quality, fatigue, anxiety/depression). Some participants (n = 62) wore an accelerometer to objectively estimate MVPA. Multiple linear regression and multivariate analysis of covariance were used to test study aims.

**Results:**

Rural BCS (n = 40, M = 61.1±8.4 years-old) performed significantly slower on Trails-B (p<0.01) compared with urban BCS (n = 40, M = 61.0±8.2 years-old) and engaged in less objectively-estimated daily MVPA (mean difference = 13.83±4.73 minutes; *p* = 0.01). No significant differences in SMI, self-reported MVPA, or CACD correlates were observed (all *p*>0.28). Regression models did not reveal a significant interaction between MVPA and cognitive performance (all *p*>0.1); however, estimated marginal means models indicated that the effect of MVPA on processing speed was evident only among rural BCS (Trails-B, *p* = 0.04; Mazes, *p* = 0.03).

**Conclusions:**

Findings suggest rural BCS may suffer greater CACD and engage in less MVPA. Additional research is warranted to further examine CACD and more effectively promote MVPA in rural BCS.

## Introduction

Compared to urban breast cancer survivors (BCS), rural BCS experience greater health consequences often associated with lower education and socioeconomic status, reduced access to health insurance, and greater geographic barriers to healthcare [1]. Rural older adults (i.e., classified by zip code or county of residence at their time of diagnosis) in the United States specifically cite out-of-town travel, service expenses, and physician recruitment and retention as the top barriers to accessing appropriate medical care, which may result in greater long-term symptom burden after cancer [2]. Studies examining cancer health disparities have identified rural cancer survivors as 62% less likely to be physically active; 1.23 times more likely to experience greater psychological distress; more likely to experience cancer-related fatigue (72.7% vs. 63.3% in urban survivors) sleep disturbances, and poor quality of life; and 1.15 times more likely to report multiple comorbid conditions (OR = 1.15) [3–7]. Despite this evidence, rural survivors remain an underrepresented population in clinical oncology research [8, 9].

Cancer-associated cognitive decline (CACD) has been reported as one of the most troubling consequences of cancer [10]. CACD is defined as cognitive impairments (i.e., 1.5–2 standard deviations below normative values on standardized neuropsychological assessments) related to cancer treatment that go beyond the effects or normal aging and is often observed as declines in processing speed, memory, and attention [11]. CACD-related difficulties reported by BCS include trouble completing habitual tasks, fulfilling social roles, concentrating, multi-tasking, and returning to work [12, 13]. Compared to women without a history of cancer, BCS also experience significantly greater perceived cognitive impairments [14]. Specifically, studies have documented declines in cognition among 75% of survivors during treatment, with 45% experiencing decline after treatment ends [15]. While some survivors report improved cognition after treatment completion, up to 30% experience persistent declines long-term [16]. Scientific frameworks of CACD have indicated that, while cancer treatments (e.g., surgery, chemotherapy, endocrine therapy, radiation therapy) can have a direct impact on cognitive function, they may also interact with psychological (e.g., anxiety, depression), physiological (e.g., biologic age, comorbid conditions, fatigue), and lifestyle factors (e.g., sleep quality, physical activity [PA] levels) that may exacerbate or provide some protection from CACD [17]. For example, theories and empirical evidence in CRCI indicate that individuals who are younger, more active, and have greater cognitive reserve may suffer the fewest cognitive consequences, while the those who are older, less active, and have limited cognitive reserve may experience greater and more permanent cognitive impairment [18, 19].

Importantly, many of these CACD determinants are also cancer-related health factors evidenced to differ between rural and urban survivors (e.g., poor sleep quality, fatigue, anxiety, depressive symptoms, PA) [2, 5]. Evidence indicates that rural older adults, compared with those living in urban/suburban areas, have a higher risk of developing cognitive dysfunction and Alzheimer’s disease [20]. This literature also demonstrates that rural older adults perform worse in objective measures of global cognitive function, episodic memory, attentional capacity, and executive function [21]. The intersection between cancer and aging (i.e., median age of cancer diagnosis in United States is 66 years-old; [22]) results in a vulnerable population of adults who may be subject to an accelerated version of age-related cognitive decline [23]. As such, rural survivors, particularly older survivors, may experience greater declines in cognitive function as compared to their urban counterparts. However, no studies, to our knowledge, have examined cognitive function among rural cancer survivors.

PA is an evidenced-based behavioral treatment for age-related cognitive decline [24] and CACD correlates in cancer survivors (e.g., fatigue) [25], with growing evidence in relation to CACD specifically [26–28]. Meta-analyses in older adults have reported moderate (g = 0.35; [24]) to large effect sizes (*g* = 0.67; [29]) of the association between aerobic PA and cognitive function. Recent proof-of-concept trials in BCS have observed improved performance on processing speed tasks among BCS randomized to a PA intervention compared to control [30, 31]. For example, Hartman and colleagues (2018) observed dose-response associations in which a 15-minute per day increase in moderate-to-vigorous PA (MVPA) among BCS was associated with improved processing speed performance and self-reported cognition [31]. Among rural survivors, self-reported PA has been associated with higher self-rated health status [6] and better physical functioning, vitality, social functioning, and emotional well-being [32], all known correlates of cognitive function [33]. However, no data on cognitive function in rural survivors are available. Previous PA studies have documented significantly less self-reported MVPA among rural survivors compared with urban survivors [5, 6]. In fact, Mama and colleagues (2020) found that urban survivors were 2.6 times more likely than rural survivors to meet aerobic PA recommendations [6]. Current evidence suggesting greater symptom burden and lower PA levels in rural cancer survivors, coupled with emerging evidence on CACD more broadly, warrant efforts to better understand and target CACD and PA in rural cancer survivors.

The purpose of this study was to examine differences in cognitive function (i.e., processing speed, working memory, subjective memory impairment [SMI]), PA (self-reported leisure-time PA and accelerometer-derived objectively-estimated MVPA), and other CACD correlates (i.e., fatigue, anxiety, depression, quality of life, sleep quality) in rural versus urban BCS. We hypothesized that cognitive function, PA, and sleep quality would be significantly lower and fatigue, anxiety, and depression significantly higher in rural BCS when compared with age- and education-matched urban BCS. We also tested the main and interaction effects of MVPA on cognitive function between rural and urban survivors. We hypothesized that MVPA would be positively associated with cognitive function across the sample, and this relationship would be more salient among rural BCS.

## Methods

### Participants and procedures

Participants were 80 BCS aged 38–79 years (M_age_ = 61.01±8.37) drawn from a larger study. Data presented herein represent cross-sectional, baseline data from the larger, prospective observational study of 414 BCS. This study was approved by the University of Illinois at Urbana-Champaign Institutional Review Board. Details regarding recruitment and characteristics of the full sample have been previously published [26, 34, 35]. Briefly, eligible participants were women aged 21 years and older who were diagnosed with breast cancer at any stage and had access to an iPad with iOS 6.1 or later and the internet. iPads used as part of this study were participants’ personal devices. Previous studies have shown that the use of personal devices for research reduces the impact of learning new systems to improve data validity [36].

Participants in the present study were recruited from July to September 2015 through the Dr. Susan Love Foundation Army of Women (now called Love Research Army), social media, BreastCancerTrials.org, and electronic fliers. Interested individuals enrolled in the study by downloading an iPad application powered by BrainBaseline^©^ (Digital Artifacts, Iowa City, IA). Participants confirmed their eligibility and signed the IRB-approved electronic informed consent via the app. Participants completed all questionnaires and cognitive tasks via the app. A subsample (n = 62) also agreed to wear an accelerometer for seven consecutive days. Rural-Urban Commuting Area (RUCA) codes (version 2.0), a four-tier scheme that incorporates population size, population density, and daily commuting patterns and is recommended for studies analyzing health status indicators as a function of access to urban-based services, were used to delineate rural and urban residence [37, 38]. Consistent with previous studies [39], zip code approximation was used to match participants’ self-reported zip codes to the appropriate RUCA code and divide the cohort into urban residence (i.e., urban core and suburban) and rural residence (i.e., large rural, small/isolated rural). BCS included in the present study were significantly older at diagnosis (Mean difference = 4.25±1.17, *p*<0.01) and at time of data collection (Mean difference = 3.25±1.13, *p*<0.01) than BCS from the larger study not included in the present sample [34]. No other differences were observed between the present sample and the total sample.

### Cognitive measures

Cognitive function was operationalized as processing speed, working memory, and SMI. Processing speed and working memory were measured using standard cognitive tasks administered via the BrainBaseline^©^ iPad application, including: Trails-B, Mazes, Task-Switch, and Spatial Working Memory (SPWM). BrainBaseline^©^ was developed by cognitive psychologists and has been validated across age groups [40]. This accessible, remote-based platform for cognitive testing has also been employed in studies of older adults, cancer patients, and individuals diagnosed with HIV to measure cognitive function across time [26, 41, 42]. SMI was assessed via a validated questionnaire.

Processing speed was operationalized as completion time on Trails-B, drawing time on Mazes, and reaction time on the Task-Switch switch trials. Trails-B required participants to draw a line as quickly as possible between a series of numbers and letters [43]. Similarly, the Mazes task was evaluated as time for participants to draw a line with their finger from the start of the maze to the finish. Task-switch measured the ability to switch between two tasks [44]. Participants were presented with a blue or pink square with a number (1–4 or 6–9) and instructed to choose if the number was higher or lower than 5 (blue square) or odd or even (pink square). Average reaction time across trials in which the color switched from blue to pink or vice versa and the participant provided a correct response was used as a measure of processing speed. Reaction time was recoded to missing for participants with less than 50% accuracy across switch trials, as this suggests the individual may have misunderstood the task instructions.

Working memory was operationalized as accuracy on the SPWM set size 3 trials [45]. On SPWM participants were shown an image of three dots, asked to remember the locations of the dots, and then asked to determine whether a delayed probe matched the location of one of the dots previously presented. The percentage of correct responses was used as a measure of working memory. Consistent with Task-Switch, data were recoded to missing if the participant had less than 50% accuracy across SPWM trials.

The Frequency of Forgetting Scale (FOF) was used to assess SMI. The FOF examines participant’s perceptions of their memory in four subscales: general memory, frequency of forgetting, frequency of forgetting while reading, and remembering past events [46, 47]. Lower scores are indicative of more memory impairment. Additional details of the cognitive tasks and FOF have been previously reported [26, 34].

### Physical activity measures

Self-reported PA was assessed using the Godin Leisure-Time Exercise Questionnaire (GLTEQ) [48]. Participants were asked to report the number of times in the past week they participated in strenuous, moderate, or mild exercise for 15 minutes or more. Frequencies for moderate and strenuous exercise were multiplied by 5 and 9 metabolic equivalents, respectively, to calculate a PA score. This PA measure has been previously validated in BCS [49].

Participants were invited to wear an accelerometer (Actigraph GT3X, Pensacola, FL) to objectively measure their PA, but this portion of the study was not required. Of the 80 participants in this sample, 62 agreed to wear the accelerometer (n_rural_ = 29, n_urban_ = 33). Participants were asked to wear the device on their non-dominant hip during waking hours for seven consecutive days. Those with ten hours of wear time for at least four days were retained for analysis [50]. Devices were initialized to capture movement in one-second epochs, and data were scored using Freedson cut points and represented as average daily minutes of MVPA (1952+ counts/minute) [51]. Freedson cut points were chosen for this analysis as they are the most commonly used cut points to examine MVPA in cancer survivors and, therefore, allow for comparison across studies [52, 53].

### Measures of CACD correlates

Demographic and clinical correlates of CACD (i.e., age, race, education, employment status, diagnosis stage, time since diagnosis, receipt of chemotherapy, months of hormonal therapy, history of depression) were also assessed via demographics and health history questionnaires [26, 31]. Major psychosocial and behavioral correlates identified in the CACD literature (i.e., fatigue, anxiety, depression, quality of life, sleep quality; [17]) were assessed via validated questionnaires. The Functional Assessment of Cancer Therapy-Fatigue Scale (FACIT-F) was used to measure cancer-related fatigue. The FACIT-F assesses self-reported fatigue and its impact on daily functioning, with lower scores indicating greater fatigue [54]. Anxiety and depressive symptoms were measured with the Hospital Anxiety and Depression Scale (HADS). The 14-item scale yields two scores, representing anxiety and depression separately, with higher scores indicating more symptoms [55]. Health-related quality of life was assessed with the functional, emotional, and physical well-being subscales of the Functional Assessment of Cancer Therapy-Breast Scale (FACT-B) [56, 57]. Higher scores on the FACT-B indicate greater quality of life. Sleep quality was operationalized as the global sleep score from the Pittsburgh Sleep Quality Index (PSQI). Higher scores are indicative of greater sleep difficulties [58].

### Data analysis

From the parent study, 40 (10%) BCS reported living in a rural area. This proportion is similar to previous studies examining rural-urban health disparities and PA in cancer survivors [6]. In the present study, a random sample of urban BCS (n = 364, 88%) were one-to-one matched to the rural subsample by age, education level, and time since diagnosis, resulting in 80 women eligible for analysis. Age, education, and time since diagnosis are frequently linked with CACD and were therefore used as matching variables and covariates [14, 15, 59].

Multiple linear regression was used to analyze associations between rurality and cognitive outcomes, MVPA, and CACD correlates. The first step of the model included demographic and clinical covariates (age, time since diagnosis, diagnosis stage, receipt of chemotherapy, months of hormonal therapy, and history of depression). The second step included rurality (rural vs. urban). Results are reported using standardized beta-coefficients and variance explained (i.e., *R*^*2*^).

For MVPA analyses, the second step of the regression model included rurality, MVPA modeled continuously from GLTEQ or accelerometer, and the interaction term. In order to interpret MVPA analyses relative to public health recommendations for PA engagement, we also modeled MVPA categorically (active v. insufficiently active). Participants were dichotomized in accordance with the 2018 Physical Activity Guidelines for Americans and 2018 American College of Sports Medicine Roundtable on Exercise Guidelines for Cancer Survivors [25, 60]. As such, participants were categorized based on self-reported MVPA using GLTEQ published thresholds for sufficiently active (≥24 units) or insufficiently active (<24 units) [61]. Those who wore the accelerometer were categorized as meeting versus not meeting PA guidelines expressed as an average of 30+ vs. <30 minutes of MVPA per day. Rurality and MVPA were centered prior to computing the interaction term to eliminate collinearity between the interaction term and the main effects.

Multivariate analysis of covariance (MANCOVA) with estimated marginal means (EMM) models was also used to explore pairwise comparisons between the rurality and MVPA category interaction on cognitive outcomes. Cognitive performance variables (Trails-B, Mazes, Task-Switch, SPWM) were included as the dependent variables in one multivariate model, and FOF total score was modeled separately in a univariate model. Pairwise comparisons based upon rurality and MVPA category in the EMM models were analyzed with a Bonferroni correction to account for multiple comparisons. Effect sizes are reported as partial eta squared (partial eta^2^). All outcome variables were Winsorized at 3 standard deviations from the mean prior to all data analyses. A type 1 error rate of α = 0.05 was used to determine statistical significance. Data were analyzed using IBM SPSS Statistics, Version 27 (SPSS Corp., Armonk, NY, USA).

## Results

Participants were, on average, educated, affluent, Caucasian, married, and overweight BCS with relatively low symptom burden (Table 1). Most women were diagnosed with early-stage breast cancer and treated with radiation therapy, chemotherapy, or both. Urban BCS reported longer duration of hormonal therapy (*p* = 0.02; Table 1). No other significant demographic or clinical differences were observed between rural and urban BCS. Women who elected to wear the accelerometer were significantly older than those who did not (*p* = 0.04).

**Table 1:** Participant characteristics.

		Rural (n = 40)		Urban (n = 40)		Total (N = 80)	
		M	±SD^a^	M	±SD	M	±SD
		n	(%)	n	(%)	n	(%)
**Demographics**							
Age (years)*		61.05	±8.40	61.00	±8.24	61.03	±8.27
Bachelor’s Degree		28	(70.0)	26	(65.0)	54	(67.5)
Income ≥ $75,000 per year		30	(81.1)	30	(81.1)	60	(78.9)
Employed full-time		13	(32.5)	10	(25.0)	23	(28.7)
Retired		18	(45.0)	19	(47.5)	37	(46.3)
White		40	(100.0)	38	(95.0)	76	(97.4)
Married		32	(80.0)	32	(80.0)	64	(80.0)
Body mass index (kg/m^2^)		26.55	±4.41	25.87	±4.69	26.20	±4.53
**Clinical**							
Cancer Stage							
	0	5	(12.5)	4	(10.0)	9	(11.3)
	1	18	(45.0)	17	(42.5)	35	(43.8)
	2	10	(25.0)	12	(30.0)	22	(27.5)
	3	7	(17.5)	5	(12.5)	12	(15.0)
	4	0	(0.0)	2	(5.0)	2	(2.5)
Estrogen receptor positive		31	(77.5)	33	(82.5)	64	(80.0)
Menopausal at diagnosis		26	(65.0)	23	(57.5)	49	(61.3)
Months since diagnosis		103.13	±80.76	106.71	±83.05	104.87	±81.37
Chemotherapy only		9	(22.5)	11	(27.5)	20	(25.0)
Radiation only		8	(20.0)	10	(25.0)	18	(22.5)
Chemotherapy and radiation		20	(50.0)	15	(37.5)	35	(43.8)
Hormonal therapy (months)*		13.55	±24.92	29.20	±34.96	21.37	±31.18
Diagnosed with Depression		10	(25.0)	4	(10.0)	14	(17.5)
**Physical Activity**							
GLTEQ (24+)		20	(50.0)	25	(62.5)	45	(56.3)
GLTEQ Score		22.95	±20.63	26.16	±16.21	24.53	±18.53
MVPA (30+ min)		8	(27.6)	16	(48.5)	24	(38.7)
MVPA (average daily min)**		20.96	±15.16	33.67	±22.68	27.72	±20.40

^a^Mean, Standard Deviation

*Significant between group differences, *p*<0.05

**Significant between group difference, *p*<0.01

### Associations between rurality and cognitive outcomes, MVPA, and CACD correlates

#### Cognitive outcomes

The regression model including only covariates was significantly associated with Trails-B completion time and explained 23.5% of the variance in performance (*F*(6,63) = 3.23, *p* = 0.008). Only age was significantly associated with Trails-B performance (*β* = 0.29, *p* = 0.02), and receipt of chemotherapy approached significance (*β* = -0.24, *p* = 0.06). Specifically, younger age and receipt of chemotherapy were associated with faster Trails-B performance. In addition, the covariates model was associated with total FOF score (*F*(6,69) = 3.44, *p* = 0.005) and explained 23% of the variance in the score. Only history of depression was significantly associated with FOF score (*β* = -0.35, *p* = 0.002) in which women diagnosed with depression reported more SMI. The covariates model was not significantly associated with Mazes drawing time (*p* = 0.34), Task-Switch reaction time (*p* = 0.10), or SPWM accuracy (*p* = 0.49). Age was independently associated with Mazes drawing time (*β* = 0.27, *p* = 0.05), with younger age associated with faster Mazes performance.

After adjustment for covariates, rurality was significantly associated with Trail-B completion time (*β* = 0.30, *p* = 0.007) and explained an additional 8.6% of the variance in Trails-B performance (Table 2). These data indicated that BCS living in rural areas had slower completion time on Trails-B. Rurality was not significantly associated with Mazes drawing time (*p* = 0.45), Task-Switch reaction time (*p* = 0.79), SPWM accuracy (*p* = 0.90), or FOF score (*p* = 0.37).

**Table 2:** MVPA, CACD correlates, and cognitive outcomes in rural and urban breast cancer survivors.

	Rural		Urban	
	M	±SD^**a**^	M	±SD
**Physical Activity**				
GLTEQ	30.48	±21.39	34.92	±20.43
MVPA*	20.96	±15.16	33.67	±22.68
Sedentary Time	586.95	±71.13	583.86	±70.76
**CACD Correlates** ^**a**^				
Fatigue	42.18	±8.87	43.25	±7.94
Anxiety	4.18	±3.57	3.73	±2.42
Depression	3.95	±3.75	2.90	±3.01
Global Sleep Score	7.32	±3.72	7.71	±3.86
Sleep Efficiency	84.62	±6.34	82.94	±6.91
**Quality of Life**				
FACT-B Total Score	112.00	±16.83	113.58	±17.33
**Subjective Memory Impairment** ^**a**^				
FOF Total Score	49.60	±9.33	49.41	±8.64
**Processing Speed** ^**b**^				
Trails-B Total Time (s)**	78.64	±38.01	62.41	±18.38
Mazes Drawing Time (s)	12.56	±3.78	13.69	±4.23
Task-Switch Reaction Time (ms)	1391.13	±158.28	1385.34	±158.43
**Working Memory** ^**b**^				
SPWM Accuracy	0.88	±0.069	0.88	±0.081

^a^Higher fatigue, FACT-B, and FOF scores denote better outcomes; Lower anxiety and depression, global sleep, and sleep efficiency scores denote better outcomes.

^b^Lower processing speed values and higher working memory values denote better cognitive performance.

*Significant difference, *p*<0.05, after adjustment for covariates (e.g., age, time since diagnosis, diagnosis stage, receipt of chemotherapy, months of hormonal therapy, and history of depression)

**Significant difference, *p*<0.01, after adjustment for covariates

#### MVPA

The regression model including only covariates was not significantly associated with self-reported MVPA (*p* = 0.08) or objectively-estimated MVPA (*p* = 0.28). However, age was independently associated with both (self-reported: *β* = -0.34, *p* = 0.01; objectively-estimated: *β* = -0.31, *p* = 0.04), with younger women engaging in more MVPA. Rurality was not significantly associated with self-reported MVPA from the GLTEQ (*p* = 0.29). However, it was associated with objectively-estimated MVPA (*β* = -0.34, *p* = 0.01) and explained 12.3% of the variance in MVPA. Rural BCS engaged in 13.83 (SE = 4.73) fewer minutes of daily MVPA compared with urban BCS.

#### CACD correlates

The covariates model was significantly associated with self-reported fatigue (*F*(6,70) = 3.20, *p* = 0.008; *R*^*2*^ = 0.22), anxiety symptoms (*F*(6,70) = 4.44, *p* = 0.001; *R*^*2*^ = 0.28), depressive symptoms (*F*(6,70) = 4.05, *p* = 0.002; *R*^*2*^ = 0.26), quality of life (*F*(6,70) = 2.70, *p* = 0.02; *R*^*2*^ = 0.19), and sleep quality (*F*(6,66) = 2.56, *p* = 0.03; *R*^*2*^ = 0.19). Specifically, history of depression was significantly associated with self-reporting poorer levels of all CACD correlates: fatigue (*β* = -0.40, *p*<0.001), anxiety symptoms (*β* = 0.38, *p* = 0.001), depressive symptoms (*β* = 0.45, *p*<0.001), quality of life (*β* = -0.34, *p* = 0.004), and sleep quality (*β* = 0.32, *p* = 0.007). Age approached significance for depressive symptoms (*β* = -0.23, *p* = 0.056), with older age being associated with lower HADS-D scores. Rurality was not significantly associated with any of the hypothesized CACD correlates (all *p*>0.28).

### Effects of MVPA on cognitive outcomes

#### Self-reported MVPA

Across the sample, self-reported, leisure-time MVPA from the GLTEQ, when modeled as a continuous variable, was not associated with performance on any of the cognitive tasks or with SMI (all *p*>0.05). However, BCS who self-reported sufficient levels of leisure-time MVPA based upon the GLTEQ performed faster on Task-Switch (*β* = -0.25, *p* = 0.049). No rurality*GLTEQ interactions were observed, and exploratory pairwise comparisons did not indicate any differences in cognitive performance or SMI.

#### Objectively-estimated MVPA

**Fig 1:**
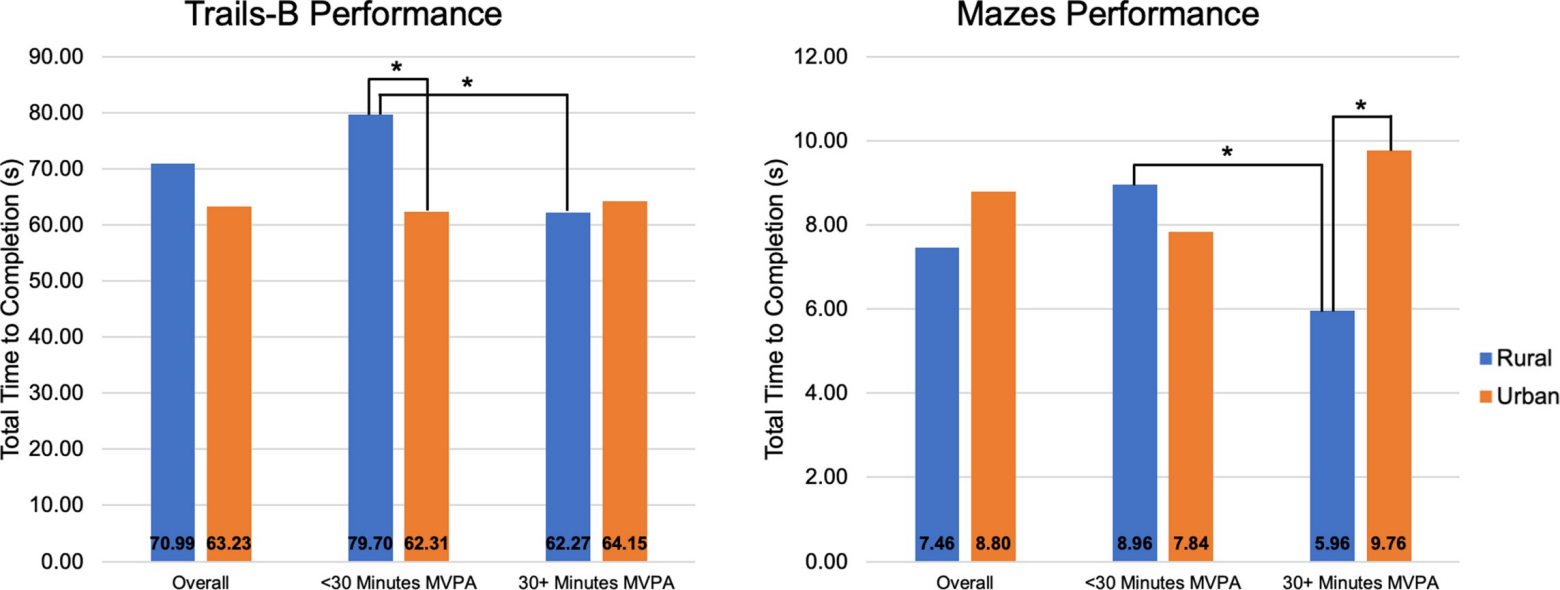
Trails-B and Mazes by rurality and MVPA status. *Significant difference, *p*<0.05. Estimated Marginal Means adjusted for age, education, diagnosis stage, time since diagnosis, receipt of chemotherapy, months of hormonal therapy, history of depression.

Across the sample, objectively-estimated MVPA, when modeled as a continuous variable, was significantly associated with Trails-B completion time (*β* = -0.32, *p* = 0.01; *R*^*2*^ = 0.09). However, meeting PA guidelines (>30 minutes per day) was not significantly associated with Trails-B (*p* = 0.085). The interaction between rurality and objectively-estimated MVPA was also not associated with Trails-B performance (*p* = 0.11). No other associations between MVPA levels or meeting PA guidelines and cognitive performance or SMI were observed (all *p*>0.21). Despite this, exploratory EMMs indicated that rural BCS who met objectively-estimated PA guidelines performed significantly faster on Trails-B (*p* = 0.04; partial eta^2^ = 0.09) and Mazes (*p* = 0.03; partial eta^2^ = 0.10) compared to rural BCS who did not meet guidelines. These differences were not observed in urban BCS (both *p*>0.11). Further, rural BCS not meeting PA guidelines (<30 minutes of daily MVPA) performed significantly slower on Trails-B when compared with urban BCS also not meeting PA guidelines (*p* = 0.01; partial eta^2^ = 0.14). Conversely, rural BCS meeting PA guidelines (30+ minutes of daily MVPA) performed significantly faster on Mazes when compared with urban BCS also meeting PA guidelines (*p* = 0.01; partial eta^2^ = 0.13). Pairwise comparisons of the rurality*objective MVPA interaction on Trails-B and Mazes are illustrated in Fig 1.

## Discussion

The present study contributes to the emerging literature on cancer health disparities in rural populations and provides new evidence on CACD. Similar to previous work, rural BCS in this study engaged in significantly less PA than their urban counterparts [6]. They also exhibited worse processing speed, one of the cognitive domains with consistent evidence of decline with cancer [10] and of improvement with regular PA [24, 31]. Interestingly, the relationship between daily MVPA and processing speed was evident only in rural BCS. Together, these findings indicate a critical need to design health promotion interventions specifically targeting rural survivors.

Independent of demographic and clinical factors, we observed significant differences in processing speed (i.e., Trails-B) between rural and urban survivors. However, no differences in working memory or SMI were observed. The cognitive performance findings mirror those in the aging literature, which posits that rural dwellers may suffer greater cognitive impairment than urban residents [62, 63]. Factors of urban life, such as complex living environments, time-pressured problem solving, leisure-time activities, and increased likelihood of personal interactions, have been identified as stimulating factors for attention, verbal memory, and visual processing [62]. As such, interventions targeting modifiable risk factors for cognitive decline (e.g., PA) may be specifically important in rural BCS.

Additional evidence suggests rural survivors also experience greater psychological distress, sleep disturbances, and fatigue [3, 4]. Rural BCS in this study were more likely to report depression diagnoses, and our analyses indicated a clinically meaningful [64], although not statistically significant, difference in depressive symptoms between rural and urban survivors. Depression is a known predictor of CACD [17], and physical activity is a well-documented treatment for depression in cancer survivors [25]. Contrary to this previous research, we did not observe rural-urban differences in any other symptoms known to be associated with CACD [17]. Previous research has observed hesitation among rural BCS to seek support for social, physical, and functional well-being, cancer-related symptoms, and quality of life [7], which may lead to biased self-report data. It is, therefore, possible that rural survivors under-reported fatigue, anxiety, depression, quality of life, and sleep quality. However, if accurately reported, these proposed CACD correlates, as our findings suggest, may not be associated with the cognitive differences observed between rural and urban BCS. PA, on the other hand, is a modifiable factor that may partially explain differences in CACD observed in the present study.

PA data from this study are representative of the current literature on PA in rural populations [5, 65–67]. Specifically, although no significant between-group differences in self-reported PA were observed, large, significant differences in objectively measured PA were evident. Previous studies have observed a similar phenomenon in rural older adults and cancer survivors [67–69]. Robertson and colleagues (2018) found no significant differences in self-reported aerobic PA between rural and urban cancer survivors, citing that rural residents may engage in greater levels of occupational, household, or transit-related PA as compared to leisure-time PA [69]. Alternatively, Mazzoni and colleagues (2017) posited that that recall bias in BCS may be higher due to cognitive impairments related to treatment [67]. In alignment with this hypothesis, rural BCS in this study exhibited lower cognitive performance and over-estimated leisure-time exercise as compared to accelerometer-derived PA data.

Consistent with previous research [5, 6, 70], rural BCS engaged in 13.13 fewer daily minutes of MVPA than urban BCS as measured by the accelerometer. Compared to previous studies examining PA guideline adherence in rural and urban cancer survivors, a greater proportion of rural BCS in this study met aerobic PA guidelines (27.6% versus 18.8% [5] and 17.1% [6]). Similarly, 48.5% of urban BCS in this study met aerobic PA guidelines as compared to 22.8% [5] and 33.5% [6]. In a recent study by Mama and colleagues (2020), urban survivors were 2.6 times more likely to meet PA recommendations compared with rural survivors, and more than half of rural survivors did not participate in any leisure-time PA [6]. This study also indicated that aerobic PA may drive rural-urban differences in total leisure-time PA. Together with the data presented in this study, these previous findings underscore the importance of developing PA interventions to reduce health disparities and improve health outcomes in rural survivors.

Importantly, engagement in regular MVPA attenuated rural-urban differences in cognitive function. Performance on processing speed tasks among rural survivors who met PA guidelines (as estimated by the accelerometer) was faster than rural BCS who did not meet guidelines and similar to that of urban BCS who did not meet guidelines. Among urban BCS, cognitive performance did not differ based on MVPA level. Further, rural BCS not meeting PA guidelines demonstrated the slowest reaction/performance times, which were significantly slower than urban BCS also not meeting PA guidelines. As such, PA may be an important intervention approach to improve cognition in rural BCS. For example, a study on rural BCS’s exercise preferences indicated that survivors may prefer unsupervised, moderate-intensity walking [70], which has been previously used as an intervention to ameliorate CACD in BCS [31]. PA interventions have effectively improved PA engagement in rural cancer survivors [8], and researchers may capitalize on this evidence to design programs targeting cognitive function and that are accessible to rural BCS facing distinct symptom burden and unique PA barriers [5].

## Conclusion

This study demonstrated that rural BCS, even when matched on age, education, and time since breast cancer diagnosis, had slower processing speed on cognitive tasks and engaged in fewer daily minutes of objectively-estimated MVPA compared with urban survivors. While differences in working memory tasks did not reach statistical significance, estimates were in the expected direction and warrant further research in a larger, more representative sample. Despite prior research in this area indicating substantial differences in sleep quality, fatigue, and psychosocial health between rural and urban cancer survivors [3, 4, 9], no differences were observed in these correlates of CACD in this sample. Instead, pairwise comparisons suggested that MVPA may be an important factor related to rural-urban differences in cognitive performance. Because rural survivors have been consistently reported as being less active than their rural counterparts, the development of accessible, PA promotion programs to help rural survivors meet PA guidelines may be critical to mitigating cognitive health disparities.

### Limitations

The present study offered several strengths (e.g., national sample, objective and subjective measures of PA and cognitive function), but also has limitations. The cross-sectional analysis prevents the evaluation of causal links among MVPA, CACD correlates, and cognitive function. Our sample was small and overwhelmingly well-educated, affluent, and white, which limits generalizability. The iPad inclusion criterion may have excluded BCS who are socioeconomically disadvantaged, socially isolated, or living in areas with no or limited internet access. While we measured a number of CACD correlates identified in the literature [17], we did not measure loneliness, social isolation, or environmental factors that could explain the cognitive differences observed. Future research may benefit from the inclusion of additional measures to improve our understanding of determinants of cognitive impairment in rural cancer populations. Overall, while results should be interpreted with caution due to study limitations, findings provide critical, early data indicating a need for more research focused on cancer health disparities in rural populations, especially regarding understudied health outcomes like PA and CACD.

## Statements and declarations

### Ethics approval

This study was approved by the University of Illinois at Urbana‐Champaign Institutional Review Board (NCT02523677).

### Consent to participate

All participants included in this study signed the electronic informed consent through the BrainBaseline^©^ application prior to participation.

## Supporting information

S1 Dataset (CSV)

## References

[1] Bergin R. J. et al., “Rural–Urban Disparities in Time to Diagnosis and Treatment for Colorectal and Breast Cancer,” Cancer Epidemiol. Biomarkers Prev., vol. 27, no. 9, pp. 1036–1046, Sep. 2018, doi: 10.1158/1055-9965.EPI-18-0210 29987098

[2] Belasco E. J., Gong G., Pence B., and Wilkes E., “The Impact of Rural Health Care Accessibility on Cancer-Related Behaviors and Outcomes,” Appl. Health Econ. Health Policy, vol. 12, no. 4, pp. 461–470, Aug. 2014, doi: 10.1007/s40258-014-0099-4 24889860

[3] Burris J. L. and Andrykowski M., “Disparities in mental health between rural and nonrural cancer survivors: A preliminary study,” Psychooncology., 2010, doi: 10.1002/pon.1600 19582800 PMC2880195

[4] Beck S. L., Towsley G. L., Caserta M. S., Lindau K., and Dudley W. N., “Symptom experiences and quality of life of rural and urban older adult cancer survivors.,” Cancer Nurs., vol. 32, no. 5, pp. 359–69, Sep. 2009, doi: 10.1097/NCC.0b013e3181a52533 19661799

[5] Weaver K. E., Palmer N., Lu L., Case L. D., and Geiger A. M., “Rural-urban differences in health behaviors and implications for health status among US cancer survivors.,” Cancer Causes Control, vol. 24, no. 8, pp. 1481–90, Aug. 2013, doi: 10.1007/s10552-013-0225-x 23677333 PMC3730816

[6] Mama S. K. et al., “Rural-urban differences in meeting physical activity recommendations and health status in cancer survivors in central Pennsylvania,” Support. Care Cancer, vol. 28, no. 10, pp. 5013–5022, Oct. 2020, doi: 10.1007/s00520-020-05342-y 32036469 PMC7415488

[7] Reid-Arndt S. A. and Cox C. R., “Does rurality affect quality of life following treatment for breast cancer?,” J. Rural Heal., 2010, doi: 10.1111/j.1748-0361.2010.00295.x 21029176 PMC2967464

[8] Bhuiyan N., Singh P., Harden S. M., and Mama S. K., “Rural physical activity interventions in the United States: A systematic review and RE-AIM evaluation,” International Journal of Behavioral Nutrition and Physical Activity, vol. 16, no. 1. BioMed Central Ltd., p. 140, Dec. 27, 2019. doi: 10.1186/s12966-019-0903-5 31882013 PMC6935185

[9] Weaver K. E., Geiger A. M., Lu L., and Case L. D., “Rural-urban disparities in health status among US cancer survivors.,” Cancer, vol. 119, no. 5, pp. 1050–7, Mar. 2013, doi: 10.1002/cncr.27840 23096263 PMC3679645

[10] Wefel J. S., Kesler S. R., Noll K. R., and Schagen S. B., “Clinical characteristics, pathophysiology, and management of noncentral nervous system cancer-related cognitive impairment in adults,” CA. Cancer J. Clin., vol. 65, no. 2, pp. 123–138, Mar. 2015, doi: 10.3322/caac.21258 25483452 PMC4355212

[11] Wefel J. S., Vardy J., Ahles T., and Schagen S. B., “International Cognition and Cancer Task Force recommendations to harmonise studies of cognitive function in patients with cancer,” Lancet Oncol., vol. 12, no. 7, pp. 703–708, Jul. 2011, doi: 10.1016/S1470-2045(10)70294-1 21354373

[12] Henneghan A., “Modifiable factors and cognitive dysfunction in breast cancer survivors: a mixed-method systematic review,” Support. Care Cancer, vol. 24, no. 1, pp. 481–497, Jan. 2016, doi: 10.1007/s00520-015-2927-y 26416490

[13] Lange M. and Joly F., “How to identify and manage cognitive dysfunction after breast cancer treatment,” J. Oncol. Pract., vol. 13, no. 12, pp. 784–790, Dec. 2017, doi: 10.1200/JOP.2017.026286 29232539

[14] Janelsins M. C., Kesler S. R., Ahles T. A., and Morrow G. R., “Prevalence, mechanisms, and management of cancer-related cognitive impairment.,” Int. Rev. Psychiatry, vol. 26, no. 1, pp. 102–13, Feb. 2014, doi: 10.3109/09540261.2013.864260 24716504 PMC4084673

[15] Janelsins M. C. et al., “Cognitive complaints in survivors of breast cancer after chemotherapy compared With Age-Matched Controls: An analysis from a nationwide, multicenter, prospective longitudinal study,” in Journal of Clinical Oncology, Feb. 2017, vol. 35, no. 5, pp. 506–514. doi: 10.1200/JCO.2016.68.5826 28029304 PMC5455314

[16] Koppelmans V., Breteler M. M. B., Boogerd W., Seynaeve C., Gundy C., and Schagen S. B., “Neuropsychological Performance in Survivors of Breast Cancer More Than 20 Years After Adjuvant Chemotherapy,” J. Clin. Oncol., vol. 30, no. 10, pp. 1080–1086, 2012, doi: 10.1200/JCO.2011.37.0189 22370315

[17] Ahles T. A. and Root J. C., “Cognitive Effects of Cancer and Cancer Treatments,” Annu. Rev. Clin. Psychol., vol. 14, no. 1, pp. 425–451, May 2018, doi: 10.1146/annurev-clinpsy-050817-084903 29345974 PMC9118140

[18] M. Mackenzie, K. Zuniga, and E. McAuley, “Cognitive impairment in breast cancer: The protective role of physical activity, cardiorespiratory fitness, and exercise training.,” in Exercise-Cognition Interactinn: Neuroscience Perspectives, T. McMorris, Ed. Elsevier, 2016, pp. 399–419.

[19] Salerno E. A. et al., “Physical Activity Patterns and Relationships With Cognitive Function in Patients With Breast Cancer Before, During, and After Chemotherapy in a Prospective, Nationwide Study,” J. Clin. Oncol., vol. 39, no. 29, pp. 3283–3292, Oct. 2021, doi: 10.1200/JCO.20.03514 34406822 PMC8500586

[20] Russ T. C., Batty G. D., Hearnshaw G. F., Fenton C., and Starr J. M., “Geographical variation in dementia: systematic review with meta-analysis.,” Int. J. Epidemiol., vol. 41, no. 4, pp. 1012–32, Aug. 2012, doi: 10.1093/ije/dys103 22798662 PMC3429875

[21] Falck R. S., Wilcox S., Best J. R., Chandler J. L., and Liu-Ambrose T., “The Association Between Physical Performance and Executive Function in a Sample of Rural Older Adults from South Carolina, USA,” Exp. Aging Res., 2017, doi: 10.1080/0361073X.2017.1276379 28230419

[22] Siegel R. L., Miller K. D., Fuchs H. E., and Jemal A., “Cancer Statistics, 2021,” CA. Cancer J. Clin., vol. 71, no. 1, pp. 7–33, Jan. 2021, doi: 10.3322/caac.21654 33433946

[23] Mandelblatt J. S. et al., “Long-term trajectories of self-reported cognitive function in a cohort of older survivors of breast cancer: CALGB 369901 (Alliance).,” Cancer, vol. 122, no. 22, pp. 3555–3563, Nov. 2016, doi: 10.1002/cncr.30208 27447359 PMC5113662

[24] Northey J. M., Cherbuin N., Pumpa K. L., Smee D. J., and Rattray B., “Exercise interventions for cognitive function in adults older than 50: a systematic review with meta-analysis.,” Br. J. Sports Med., vol. 52, no. 3, pp. 154–160, Feb. 2018, doi: 10.1136/bjsports-2016-096587 28438770

[25] Campbell K. L. et al., “Exercise Guidelines for Cancer Survivors: Consensus Statement from International Multidisciplinary Roundtable,” Med. Sci. Sports Exerc., vol. 51, no. 11, pp. 2375–2390, Nov. 2019, doi: 10.1249/MSS.0000000000002116 31626055 PMC8576825

[26] Ehlers D. K., Aguiñaga S., Cosman J., Severson J., Kramer A. F., and McAuley E., “The effects of physical activity and fatigue on cognitive performance in breast cancer survivors,” Breast Cancer Res. Treat., vol. 165, no. 3, pp. 699–707, 2017, doi: 10.1007/s10549-017-4363-9 28677009 PMC12257920

[27] Ehlers D. K., Rogers L. Q., Courneya K. S., Robbs R. S., and McAuley E., “Effects of BEAT Cancer randomized physical activity trial on subjective memory impairments in breast cancer survivors,” Psychooncology., vol. 27, no. 2, pp. 687–690, 2018, doi: 10.1002/pon.4438 28414894 PMC5643210

[28] Hartman S. J. et al., “Randomized controlled trial of increasing physical activity on objectively measured and self-reported cognitive functioning among breast cancer survivors: The memory & motion study,” Cancer, pp. 1–11, 2017, doi: 10.1002/cncr.30987 28926676 PMC5735009

[29] Colcombe S. J. et al., “Aerobic Fitness Reduces Brain Tissue Loss in Aging Humans,” Journals Gerontol. Ser. A Biol. Sci. Med. Sci., vol. 58, no. 2, pp. M176–M180, 2003, doi: 10.1093/gerona/58.2.m176 12586857

[30] Campbell K. L., Zadravec K., Bland K. A., Chesley E., Wolf F., and Janelsins M. C., “The Effect of Exercise on Cancer-Related Cognitive Impairment and Applications for Physical Therapy: Systematic Review of Randomized Controlled Trials,” Phys. Ther., vol. 100, no. 3, Mar. 2020, doi: 10.1093/ptj/pzz090 32065236 PMC8559683

[31] Hartman S. J. et al., “Randomized controlled trial of increasing physical activity on objectively measured and self-reported cognitive functioning among breast cancer survivors: The memory & motion study,” Cancer, vol. 124, no. 1, pp. 192–202, Jan. 2018, doi: 10.1002/cncr.30987 28926676 PMC5735009

[32] Rafie C., Ning Y., Wang A., Gao X., and Houlihan R., “Impact of physical activity and sleep quality on quality of life of rural residents with and without a history of cancer: Findings of the day and night study,” Cancer Manag. Res., vol. 10, pp. 5525–5535, 2018, doi: 10.2147/CMAR.S160481 30519100 PMC6234991

[33] E. McAuley and K. S. Morris, “Advances in Physical Activity and Mental Health: Quality of Life,” 10.1177/1559827607303243, vol. 1, no. 5, pp. 389–396, Oct. 2007, doi: 10.1177/1559827607303243.

[34] Ehlers D. K., Fanning J., Sunderlage A., Severson J., Kramer A. F., and McAuley E., “Influence of sitting behaviors on sleep disturbance and memory impairment in breast cancer survivors,” Cancer Med., vol. 9, no. 10, pp. 3417–3424, May 2020, doi: 10.1002/cam4.3008 32202706 PMC7221435

[35] Hanson L. L., Severson J., Kramer A. F., Mcauley E., and Ehlers., “Differences in cognition and physical activity in younger versus older breast cancer survivors,” Psychooncology., 2020. 10.1002/pon.5388 32281167

[36] Ehlers D. K., Huberty J., Buman M., Hooker S., Todd M., and de Vreede G.-J., “A Novel Inexpensive Use of Smartphone Technology for Ecological Momentary Assessment in Middle-Aged Women,” J. Phys. Act. Heal., vol. 13, no. 3, pp. 262–268, 2016, doi: 10.1123/jpah.2015-0059 26284689

[37] Hall S. A., Kaufman J. S., and Ricketts T. C., “Defining urban and rural areas in U.S. epidemiologic studies,” Journal of Urban Health. 2006. doi: 10.1007/s11524-005-9016-3 16736366 PMC2527174

[38] Washington State Department of Health, “Guidelines for Using Rural-Urban Classification Systems for Public Health Assessment,” pp. 1–26, 2001.

[39] Gray M. S., Judd S. E., Sloane R., Snyder D. C., Miller P. E., and Demark-Wahnefried W., “Rural–urban differences in health behaviors and outcomes among older, overweight, long-term cancer survivors in the RENEW randomized control trial,” Cancer Causes Control, vol. 30, no. 4, pp. 301–309, 2019, doi: 10.1007/s10552-019-01141-x 30783858 PMC6459722

[40] Lee H. et al., “Examining cognitive function across the lifespan using a mobile application,” 2012, doi: 10.1016/j.chb.2012.05.013

[41] Rubin L. H. et al., “Tablet-Based Cognitive Impairment Screening for Adults With HIV Seeking Clinical Care: Observational Study,” JMIR Ment. Heal., vol. 8, no. 9, Sep. 2021, doi: 10.2196/25660 34499048 PMC8461534

[42] Clark R., Tahan A. C., Watson P. D., Severson J., Cohen N. J., and Voss M., “Aging affects spatial reconstruction more than spatial pattern separation performance even after extended practice,” Hippocampus, vol. 27, no. 6, pp. 716–725, Jun. 2017, doi: 10.1002/hipo.22727 28321961

[43] Tombaugh T. N., “Trail Making Test A and B: Normative data stratified by age and education,” Arch. Clin. Neuropsychol., vol. 19, no. 2, pp. 203–214, Mar. 2004, doi: 10.1016/S0887-6177(03)00039-8 15010086

[44] Monsell S., “Task switching,” Trends in Cognitive Sciences, vol. 7, no. 3. Elsevier Ltd, pp. 134–140, Mar. 01, 2003. doi: 10.1016/s1364-6613(03)00028-7 12639695

[45] Awh E., Jonides J., and Reuter-Lorenz P. A., “Rehearsal in spatial working memory.,” J. Exp. Psychol. Hum. Percept. Perform., vol. 24, no. 3, pp. 780–790, 1998, doi: 10.1037//0096-1523.24.3.780 9627416

[46] Zelinski E. M. and Gilewski M. J., “A 10-item Rasch modeled memory self-efficacy scale,” Aging Ment. Health, vol. 8, no. 4, pp. 293–306, Jul. 2004, doi: 10.1080/13607860410001709665 15370046

[47] Zelinski E. M., Gilewski M. J., and Anthony-Bergstone C. R., “Memory Functioning Questionnaire: Concurrent validity with memory performance and self-reported memory failures.,” Psychol. Aging, vol. 5, no. 3, pp. 388–399, 1990, doi: 10.1037/0882-7974.5.3.388 2242243

[48] Godin G. and Shephard R., “A simple method to assess exercise behavior in the community.,” Can. J. Appl. Sport. Sci., vol. 10, no. 3, pp. 141–146, 1985. 4053261

[49] Amireault S., Godin G., Lacombe J., and Sabiston C. M., “Validation of the Godin-Shephard Leisure-Time Physical Activity Questionnaire classification coding system using accelerometer assessment among breast cancer survivors,” J. Cancer Surviv., vol. 9, no. 3, pp. 532–540, Sep. 2015, doi: 10.1007/s11764-015-0430-6 25666749

[50] Troiano R. P., Berrigan D., Dodd K. W., Mâsse L. C., Tilert T., and McDowell M., “Physical activity in the United States measured by accelerometer.,” Med. Sci. Sports Exerc., vol. 40, no. 1, pp. 181–8, Jan. 2008, doi: 10.1249/mss.0b013e31815a51b3 18091006

[51] Freedson P., Melanson E., and Sirard J., “Calibration of the Computer Science and Applications, Inc. accelerometer.,” Med. Sci. Sports Exerc., vol. 30, no. 5, pp. 777–781, 1998. doi: 10.1097/00005768-199805000-00021 9588623

[52] Peddle-Mcintyre C. J. et al., “A Review of Accelerometer-based Activity Monitoring in Cancer Survivorship Research,” Med. Sci. Sport. Exerc, vol. 50, no. 9, pp. 1790–1801, 2018, doi: 10.1249/MSS.0000000000001644 29683922

[53] Watson K. B., Carlson S. A., Carroll D. D., and Fulton J. E., “Comparison of Accelerometer Cut Points to Estimate Physical Activity in U.S. Adults,” J. Sports Sci., vol. 32, no. 7, p. 660, Apr. 2014, doi: 10.1080/02640414.2013.847278 24188163 PMC4589136

[54] Yellen S. B., Cella D. F., Webster K., Blendowski C., and Kaplan E., “Measuring fatigue and other anemia-related symptoms with the Functional Assessment of Cancer Therapy (FACT) measurement system,” J. Pain Symptom Manage., vol. 13, no. 2, pp. 63–74, Feb. 1997, doi: 10.1016/s0885-3924(96)00274-6 9095563

[55] Zigmond A. S. and Snaith R. P., “The Hospital Anxiety and Depression Scale,” Acta Psychiatr. Scand., vol. 67, no. 6, pp. 361–370, Jun. 1983, doi: 10.1111/j.1600-0447.1983.tb09716.x 6880820

[56] Cella D., Nichol M. B., Eton D., Nelson J. B., and Mulani P., “Estimating clinically meaningful changes for the functional assessment of cancer therapy—Prostate: Results from a clinical trial of patients with metastatic hormone-refractory prostate cancer,” Value Heal., vol. 12, no. 1, pp. 124–129, 2009, doi: 10.1111/j.1524-4733.2008.00409.x 18647260

[57] Brady M. J. et al., “Reliability and validity of the Functional Assessment of Cancer Therapy-Breast quality-of-life instrument.,” J. Clin. Oncol., vol. 15, no. 3, pp. 974–86, Mar. 1997. doi: 10.1200/JCO.1997.15.3.974 9060536

[58] Buysse D. J., Reynolds C. F., Monk T. H., Berman S. R., and Kupfer D. J., “The Pittsburgh Sleep Quality Index: a new instrument for psychiatric practice and research.,” Psychiatry Res., vol. 28, no. 2, pp. 193–213, 1989, doi: 10.1016/0165-1781(89)90047-4 2748771

[59] Ahles T. A. et al., “Longitudinal assessment of cognitive changes associated with adjuvant treatment for breast cancer: Impact of age and cognitive reserve,” J. Clin. Oncol., vol. 28, no. 29, pp. 4434–4440, Oct. 2010, doi: 10.1200/JCO.2009.27.0827 20837957 PMC2988635

[60] US Department of Health and Human Services, “2018 Physical Activity Guidelines,” 2018. Accessed: Nov. 26, 2018. [Online]. Available: https://health.gov/paguidelines/second-edition/report/pdf/04_C_Background_and_Key_Physical_Activity_Concepts.pdf

[61] Godin G., “The Godin-Shephard Leisure-Time Physical Activity Questionnaire,” Heal. Fit. J. Canada, vol. 4, no. 1, pp. 18–22, 2011, doi: 10.14288/HFJC.V4I1.82

[62] Georgi H. S., Frydrychova Z., Vlckova K. H., Vidovicova L., Sulc Z., and Lukavsky J., “Young-old city-dwellers outperform village counterparts in attention and verbal control tasks,” Front. Psychol., vol. 10, no. MAY, pp. 1–15, 2019, doi: 10.3389/fpsyg.2019.01224 31191412 PMC6546844

[63] Łojko D. et al., “Association of cognitive performance with the physical activity and body mass index in middle-aged and older rural inhabitants,” Eur. Rev. Med. Pharmacol. Sci., vol. 18, no. 23, pp. 3645–3652, 2014. 25535135

[64] Puhan M. A., Frey M., Büchi S., and Schünemann H. J., “The minimal important difference of the hospital anxiety and depression scale in patients with chronic obstructive pulmonary disease,” Health Qual. Life Outcomes, vol. 6, p. 46, Jul. 2008, doi: 10.1186/1477-7525-6-46 18597689 PMC2459149

[65] Fazzino T. L., Fabian C., and Befort C. A., “Change in Physical Activity During a Weight Management Intervention for Breast Cancer Survivors: Association with Weight Outcomes,” Obesity, vol. 25, no. Suppl 2, pp. S109–S115, 2017, doi: 10.1002/oby.22007 29086523 PMC5679351

[66] Mama S. K., Bhuiyan N., Smyth J. M., and Schmitz K. H., “Stress and Physical Activity in Rural Cancer Survivors: The Moderating Role of Social Support,” J. Rural Heal., vol. 36, no. 4, pp. 543–548, Sep. 2020, doi: 10.1111/jrh.12455 32472721 PMC8247162

[67] Mazzoni A.-S., Nordin K., Berntsen S., Demmelmaier I., and Igelström H., “Comparison between logbook-reported and objectively-assessed physical activity and sedentary time in breast cancer patients: an agreement study,” BMC Sport. Sci. Med. Rehabil. 2017 91, vol. 9, no. 1, pp. 1–9, Mar. 2017, doi: 10.1186/s13102-017-0072-2 28373907 PMC5376284

[68] Cleland V. et al., “Effectiveness of interventions to promote physical activity and/or decrease sedentary behaviour among rural adults: a systematic review and meta-analysis,” Obes. Rev., vol. 18, no. 7, pp. 727–741, Jul. 2017, doi: 10.1111/obr.12533 28401687

[69] Robertson M., Song J., Taylor W., Durand C., and Basen-Engquist K., “Urban-Rural Differences in Aerobic Physical Activity, Muscle Strengthening Exercise, and Screen-Time Sedentary Behavior,” J. Rural Health, vol. 34, no. 4, pp. 401–410, Sep. 2018, doi: 10.1111/jrh.12295 29451333 PMC8170852

[70] Rogers L. Q., Markwell S. J., Verhulst S., McAuley E., and Courneya K. S., “Rural breast cancer survivors: Exercise preferences and their determinants,” Psychooncology., 2009, doi: 10.1002/pon.1497 19241491

